# Estimating Risk for Death from Coronavirus Disease, China, January–February 2020

**DOI:** 10.3201/eid2606.200233

**Published:** 2020-06

**Authors:** Kenji Mizumoto, Gerardo Chowell

**Affiliations:** Georgia State University, Atlanta, Georgia, USA (K. Mizumoto, G. Chowell);; Kyoto University, Kyoto, Japan (K. Mizumoto)

**Keywords:** coronavirus, outbreak, 2019 novel coronavirus disease, COVID-19, severe acute respiratory syndrome coronavirus 2, SARS-CoV-2, case-fatality ratio, CFR, risk, death, mortality, viruses, Wuhan, Hubei, China, respiratory diseases, zoonoses

## Abstract

Since December 2019, when the first case of coronavirus disease (COVID-19) was identified in the city of Wuhan in the Hubei Province of China, the epidemic has generated tens of thousands of cases throughout China. As of February 28, 2020, the cumulative number of reported deaths in China was 2,858. We estimated the time-delay adjusted risk for death from COVID-19 in Wuhan, as well as for China excluding Wuhan, to assess the severity of the epidemic in the country. Our estimates of the risk for death in Wuhan reached values as high as 12% in the epicenter of the epidemic and ≈1% in other, more mildly affected areas. The elevated death risk estimates are probably associated with a breakdown of the healthcare system, indicating that enhanced public health interventions, including social distancing and movement restrictions, should be implemented to bring the COVID-19 epidemic under control.

Since the first case of coronavirus disease (COVID-19) was identified in December 2019 in the city of Wuhan in the Hubei Province of China, the novel virus (severe acute respiratory syndrome coronavirus 2 [SARS-CoV-2]) has continued to spread around the world, resulting in several thousand reported cases in multiple countries. In China, the cumulative number of reported deaths was 2,858 as of February 28, 2020, a figure that already dwarfed the number of persons that succumbed to severe acute respiratory syndrome during 2002–2003 (*1*).

In the context of an emerging infectious disease with pandemic potential, assessing its efficiency at spreading between humans is critical, as is determining the associated risk for death from the disease. In particular, the type and intensity of public health interventions are often set as a function of these epidemiologic metrics. In the absence of vaccines against SARS-CoV-2 or antiviral drugs for the treatment of COVID-19, the implementation of handwashing and other hygiene-related interventions, as well as nonpharmaceutical interventions such as social distancing and movement restrictions (all of which are the basic strategies available to mitigate disease spread in the population), also generate considerable pressure on the global economy (*2*).

As interventions are gradually implemented and calibrated during the course of an outbreak, early estimates of the case-fatality ratio (CFR) provide crucial information for policymakers to decide the intensity, timing, and duration of interventions. However, the assessment of epidemiologic characteristics, including the CFR, during the course of an outbreak tends to be affected by right censoring and ascertainment bias (*3*–*5*). The phenomenon of right censoring is caused by the gap in illness onset to death between the vulnerable population and the healthy population, resulting in underestimation, whereas ascertainment bias is attributable to the unreported bulk of infected persons who have mild symptoms or asymptomatic infections, potentially leading to overestimation. Assuming that ascertainment bias is consistent, we can adjust for right censoring by using established methods and available data (*6*,*7*). To assess the current severity of the epidemic in China, we derived estimates (and quantified uncertainty) of the time-delay adjusted CFR for COVID-19 for the city of Wuhan and for China excluding Wuhan, with quantified uncertainty.

## Methods

### Data Sources

We used 2 different types of epidemiologic data in our analysis. First, we extracted the daily series of confirmed cases and deaths in China from daily reports published by the respective governments of China, Hubei Province, and the city of Wuhan (*8*–*11*). Because >50% of the deaths are occurring in Hubei Province, and most of these have occurred in Wuhan, we categorized the data by geographic area: Wuhan City, Hubei Province excluding Wuhan, or China excluding Hubei Province. Diagnosis of COVID-19 relies solely on PCR testing because rapid diagnostic tests for this novel coronavirus are not widely available. Our analysis relies on epidemiologic data reported through February 11, 2020, because of the change in case definition that was announced by the government of China on February 12 (*12*).

We then obtained from several sources a total of 50 epidemiologic descriptions of patients who died from COVID-19 (*9*–*11*). After we checked for duplication and missing data, the sample size with data available was 39 patients for observed delays from illness onset to death and 33 for observed delays from hospitalization to death. We fitted a gamma distribution, an exponential distribution, and a lognormal distribution to these distributions and selected the best model based on the Akaike information criterion (AIC) (Appendix 1). The gamma distribution yielded the best fit for the distribution of delays from hospitalization to death (AIC 202.0), whereas the log-normal distribution gave the best fit for the distribution of delays from illness onset to death (AIC 263.3). On the basis of these 2 delay distributions, we incorporated the distribution of delays from hospitalization to death into the model.

### Case-Fatality Ratio

**Figure 4:**
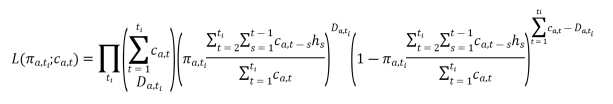
Equation for time-delay adjusted CFR.

We defined crude CFR as the number of cumulative deaths divided by the number of cumulative cases at a specific point in time. To estimate CFR in real time, we used the delay from hospitalization to death, *h*_s_, which is assumed to be given by *h*_s_ = *H*(s) – *H*(s-1) for *s*>0 where *H*(s) is a cumulative density function of the delay from hospitalization to death and follows a gamma distribution with mean 10.1 days and SD 5.4 days, obtained from the available observed data. If *π_a,ti_* is the time-delay adjusted CFR on reported day *t*i in area *a*, the likelihood function of the estimate *π_a,ti_* is (Figure 4) where *c_a,t_* represents the number of new cases with reported day *t* in area *a*, and *D_a,ti_* is the cumulative number of deaths until reported day *t*_i_ in area *a* (*6*,*7*). Among the cumulative cases with reported day *t* in area *a*, *D_a,ti_* have died, and the remainder have survived the infection. The contribution of those who have died with biased death risk is shown in the middle parenthetical term, and the contribution of survivors is shown in the right parenthetical term. We assume that *D_a,ti_* is the result of the binomial sampling process with probability *π_a,ti_*.

We estimated model parameters by using a Markov chain Monte Carlo method in a Bayesian framework. We estimated posterior distributions of the model parameters by sampling from the 3 Markov chains. For each chain, we drew 100,000 samples from the posterior distribution after a burn-in of 20,000 iterations. We evaluated convergence of Markov chain Monte Carlo chains by using the potential scale reduction statistic (*13*,*14*). Estimates and 95% credibility intervals (CrIs) for these estimates are based on the posterior probability distribution of each parameter and based on the samples drawn from the posterior distributions. All statistical analyses were conducted in R version 3.6.1 (R Foundation for Statistical Computing, https://www.r-project.org) using the rstan package.

## Results

As of February 11, 2020, a total of 44,795 cases of COVID-19 had been reported in China, 1,117 of which had resulted in death (*9**–**11*; Appendix 2). Of the 44,795 cases reported in China, 19,559 cases (43.7%) occurred in Wuhan, 13,894 cases (31.0%) occurred in Hubei Province excluding Wuhan, and 11,342 cases (25.3%) occurred in China excluding Hubei Province. Of the 1,117 deaths in China, 820 (73.4%) occurred in Wuhan, 248 (22.2%) occurred in Hubei Province excluding Wuhan, and 49 (4.4%) occurred in China excluding Hubei Province.

**Figure 1:**
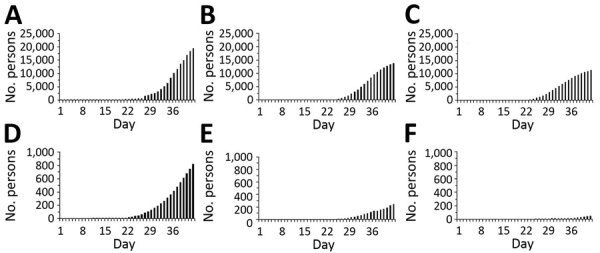
Temporal distribution of cases and deaths attributable to coronavirus disease in 3 areas in China, January 1–February 11, 2020. Cumulative cases in A) Wuhan, B) Hubei Province excluding Wuhan City, and C) China excluding Hubei Province, and cumulative deaths in D) Wuhan, E) Hubei Province excluding Wuhan, and F) China excluding Hubei Province. Day 1 corresponds to January 1, 2020. Because the dates of illness onset were not available, we used dates of reporting.

We charted the cumulative cases and deaths in Wuhan, Hubei Province excluding Wuhan, and China excluding Hubei Province (Figure 1). The curve of the cumulative number of deaths grows after that of the cumulative number of cases. Moreover, the increase in the number of deaths in Wuhan occurred more rapidly and the associated mortality rate was much higher than for the rest of China, whereas the cumulative case counts for the 3 areas in China are relatively similar.

**Figure 2:**
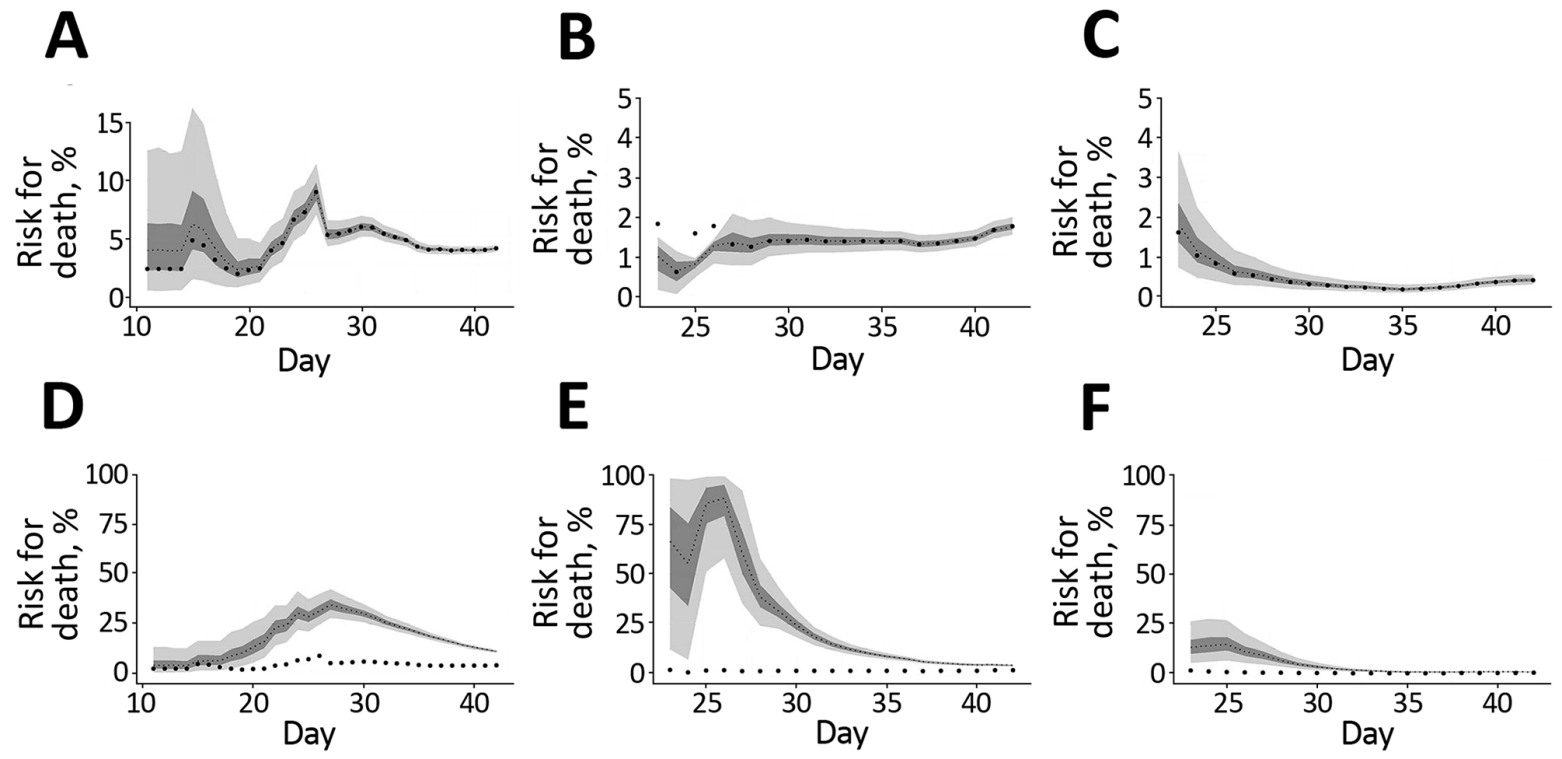
Temporal variation of risk for death associated with coronavirus disease in 3 areas in China, January 1–February 11, 2020. Observed and posterior estimates of A) crude case-fatality ratio in Wuhan, B) Hubei Province excluding Wuhan, and C) China excluding Hubei Province, and D) time-delay adjusted case-fatality ratio in Wuhan, E) Hubei Province excluding Wuhan, and F) China excluding Hubei Province. Day 1 corresponds to January 1, 2020. Black dots show crude case-fatality ratio, light gray area shows 95% credibility interval for posterior estimates, and dark gray area shows 50% credibility intervals for posterior estimates.

We also charted the observed and model-based posterior estimates of crude CFR and the model-based posterior estimates of the time-delay adjusted CFR for Wuhan, Hubei Province excluding Wuhan, and China excluding Hubei Province (Figure 2). Our model-based crude CFR fitted the observed data well throughout the course of the epidemic except for the very early stage. During the course of the outbreak, our model-based posterior estimates of time-delay adjusted CFR have much higher values than the observed crude CFR, except for the early stage in Wuhan and the later stage in China excluding Hubei Province. Our estimates of the time-delay adjusted CFR appear to be decreasing almost consistently in Hubei Province excluding Wuhan and in China excluding Hubei Province, whereas in Wuhan, estimates were low at the early stage and then increased and peaked in the middle of the study period; the Wuhan estimates then followed a decreasing trend similar to the other 2 areas, reaching ≈12%.

**Figure 3:**
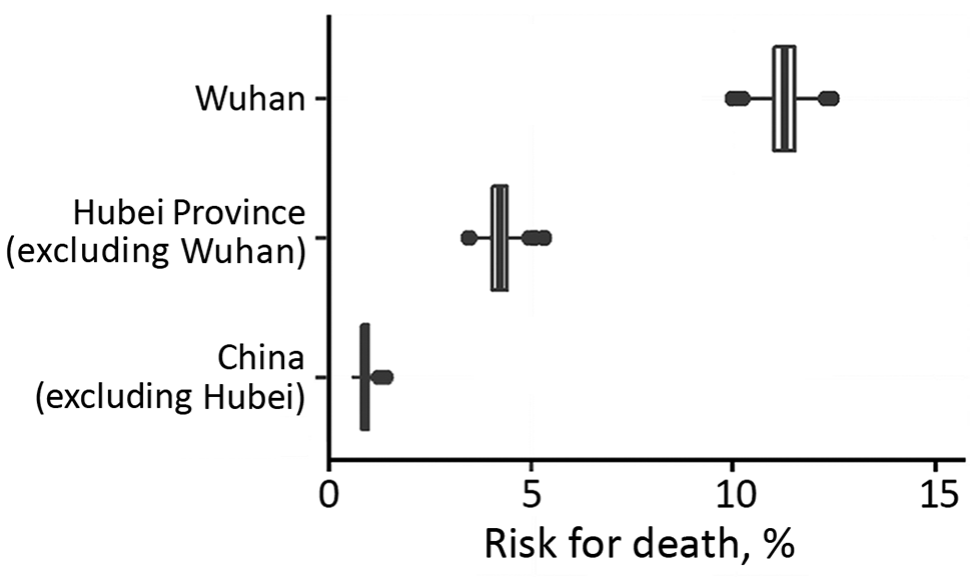
Latest estimates of time-delay adjusted risk for death from coronavirus disease in 3 areas in China, January 1–February 11, 2020.

As of February 11, estimates of the time-delay adjusted CFR were 12.2% (95% CrI 11.3%–13.1%) in Wuhan, 4.2% (95% CrI 3.7%–4.7%) in Hubei Province excluding Wuhan, and 0.9% (95% CrI 0.7%–1.1%) in China excluding Hubei Province. The observed crude CFR was 4.2% (95% CI 3.9%–4.5%) in Wuhan, 1.8% (95% CI 1.6%–2.0%) in Hubei Province excluding Wuhan, and 0.43% (95% CI 0.32%–0.57%) in China excluding Hubei Province (Table; Figure 3).

**Table:** Summary results of time-delay adjusted CFR for COVID-19 in the 3 areas in China, January 1–February 11, 2020*

Area	Latest estimate, %	Median estimates during study period, %	Crude CFR (95% CI), %	No. deaths/ no. cases
Wuhan	12.2 (95% CrI 11.3–13.1)	4.1–34.8	4.2 (95% CI 3.9–4.5)	820/19,559
Hubei Province excluding Wuhan	4.2 (95% CrI 3.7–4.7)	4.2–88.3	1.8 (95% CI 1.6–2.0)	248/13,894
China excluding Hubei Province	0.9 (95% CrI 0.7–1.1)	0.8–14.8	0.35 (95% CI 0.32–0.57)	39/11,103

*CFR, case-fatality ratio; COVID-19, coronavirus disease; CrI, credibility interval.

## Discussion

We have derived estimates of the CFR for the ongoing COVID-19 epidemic in China. We have estimated time-delay adjusted CFR in 3 different geographic areas in China and found that the most severely affected areas were Wuhan as well as Hubei Province excluding Wuhan, whereas the rest of China (China excluding Hubei Province) experienced a less severe impact.

Our latest estimates (as of February 11, 2020) of the delay-adjusted CFR in Wuhan reach values as high as 12.2% (95% CrI 11.3%–13.1%), an estimate that is 3-fold higher than our estimate for Hubei Province excluding Wuhan and ≈14-fold higher than our estimate for China excluding Hubei Province. These findings suggest that the situation in Wuhan has been particularly dire compared with the other affected areas in China. We note that the upward trend of CFR during the early phase generally indicates increasing ascertainment bias.

An upward trend in the CFR should be interpreted with caution. Diagnosing cases of COVID-19 is difficult because the associated symptoms are not specific. Further, the fraction of asymptomatic patients with SAR-CoV-2 infection and COVID-19 patients who have mild symptoms is not minor; this fact complicates detection and diagnosis early after illness onset, leading to ascertainment bias (*15*,*16*). Indeed, out of a total of 566 residents of Japan who evacuated Wuhan by government-chartered plane during January 29–31, a total of 5 asymptomatic and 4 symptomatic COVID-19 patients were detected after undergoing detailed medical examinations (*17*). However, considering that this underestimation occurred during the course of outbreak and the number of deaths is reported fairly accurately, the upward trend indicates that the temporal disease burden exceeded the capacity of healthcare facilities and the surveillance system probably missed many cases during the early phase. In addition, hospital-based transmission has occurred, potentially affecting healthcare workers, inpatients, and visitors at healthcare facilities, which might explain an increasing trend and the elevated CFR estimates. Indeed, thousands of healthcare workers have succumbed to the disease in China (*18*), a pattern that resembles past nosocomial outbreaks of Middle East respiratory syndrome (MERS) and severe acute respiratory syndrome (*19*,*20*). During past MERS outbreaks, inpatients with underlying disease or elderly persons infected in the hospital setting have raised the CFR to values as high as 20% (*21*,*22*). A growing body of evidence indicates that COVID-19 transmission is facilitated in confined settings; for example, a large cluster (634 confirmed cases) of COVID-19 secondary infections occurred aboard a cruise ship in Japan, representing about one fifth of the persons aboard who were tested for the virus. This finding indicates the high transmissibility of COVID-19 in enclosed spaces (*23*,*24*).

A downward trend in CFR is suggestive of the extent of improvements in epidemiologic surveillance. In addition, this pattern indirectly indicates a substantial number of mild or asymptomatic cases in Wuhan and that the underlying transmission might prolong the end of the outbreak or further transmission to other areas unless effective social distancing measures are implemented until a vaccine becomes available. Furthermore, given that the delay-adjusted CFR and crude CFR estimates in Wuhan are ≈14-fold higher than our estimates for China excluding Hubei Province, a breakdown in healthcare delivery probably occurred, underscoring the critical need for urgent medical support in the epicenter of the epidemic.

We also found that the estimates of the delay-adjusted CFR for Hubei Province excluding Wuhan and for China excluding Hubei Province showed a declining trend as the epidemic progressed. A similar trend was previously reported for the 2015 MERS outbreak in South Korea, where a substantial fraction of the case-patients were elderly or had underlying conditions (*19*,*20*). The high proportion of vulnerable case-patients at the early phase of the outbreak and the smaller number in the later stage could partly explain the observed decline. However, because the epidemic had yet to peak, this time-dependent decrease was probably caused by ascertainment bias. Moreover, the latest estimates of the delay-adjusted CFR and crude CFR in Hubei Province are ≈5-fold higher than our estimate for China excluding Hubei Province, where the healthcare system has not been overwhelmed. These findings also indicate the need to anticipate additional medical support to deliver medical care to the most vulnerable patients, including those with preexisting health conditions, who are at the highest risk for succumbing to the disease. For comparison, the crude CFR has been estimated at 0.9% in Beijing (*25*), 1.4% among 1,099 patients across China (*26*), and 4.3% in a meta-analysis among 50,466 hospitalized patients (*27*).

Our study has limitations. First, our CFR estimate is influenced by ascertainment bias, which might influence estimates upward. For those infectious diseases characterized by a large fraction of patients with mild illness or asymptomatic infections, the infection-fatality risk (e.g., the number of deaths divided by the total number of persons infected) is a more appropriate index of disease burden (*28*,*29*). Therefore, mass serologic surveillance and surveys to assess the presence or absence of symptoms is strongly recommended to disentangle the threat of emerging infectious diseases, including COVID-19. In addition, because our estimates of CFR are based on the number of confirmed cases reported before the February 12 change in the case definition, caution will be needed when comparing our estimates with other CFR estimates that include epidemiologic data from on or after February 12, which would be lower. Second, in our estimation we employed a distribution of delays from illness onset to death (n = 39 patients), which was obtained from secondary sources, but the available epidemiologic data does not include either the date of illness onset or the date of confirmation. For this reason, we used the time delay from hospitalization to death (n = 33 patients).

In conclusion, our estimates of the risk for death from COVID-19 in China as of February 11, 2020, were as high as 12% in the epicenter of the epidemic and as low as ≈1% in the less severely affected areas in China. Because the risk for death from COVID-19 is probably associated with a breakdown of the healthcare system in the absence of pharmaceutical interventions (i.e., vaccination and antiviral drugs), enhanced public health interventions (including social distancing measures, quarantine, enhanced infection control in healthcare settings, and movement restrictions), as well as enhanced hygienic measures in the general population and an increase in healthcare system capacity, should be implemented to rapidly contain the epidemic.

Appendix 1 Additional information regarding estimating risk for death from COVID-19, China, January–February 2020.

Appendix 2 Additional datasheets on cases and deaths attributable to COVID-19, China, January–February 2020.
