# Supplementary material for: Estimating Risk for Death from Coronavirus Disease, China, January–February 2020
Source: Emerg Infect Dis. 2020 Jun;26(6):1251–6. doi: 10.3201/eid2606.200233 (PMC7258458; doi:10.3201/eid2606.200233)
Supplement: Appendix 1 — Additional information regarding estimating risk for death from COVID-19, China, January–February 2020. [file 20-0233-Techapp-s1.pdf]

Article DOI: https://doi.org/10.3201/eid2606.200233

Estimating Risk for Death from 2019 Novel Coronavirus Disease, China, January–February 2020

Appendix 1

Appendix 1 Table 1. Delay distribution from hospitalization to death, China, 2020*

Distribution	Estimate	AIC
Gamma	Mean 10.1<br>SD 5.4	202.0
Exponential	Rate 0.10	220.6
Lognormal	Meanlog 2.16<br>SDlog 0.57	202.7

*Gamma, exponential and exponential distribution were fitted to the available observed data, and estimated parameters and AIC for each distribution are presented (sample size 33 patients). AIC, Akaike information criterion.

Appendix 1 Table 2. Delay distribution from illness onset to death, China, 2020*

Distribution	Estimate	AIC
Gamma	Mean 16.0<br>SD 8.0	269.9
Exponential	Rate 0.063	296.0
Lognormal	Meanlog 2.63<br>SDlog 0.48	263.3

*Gamma, exponential and exponential distribution were fitted to the available observed data, and estimated parameters and AIC for each distribution are presented (sample size 39 patients). AIC, Akaike information criterion.

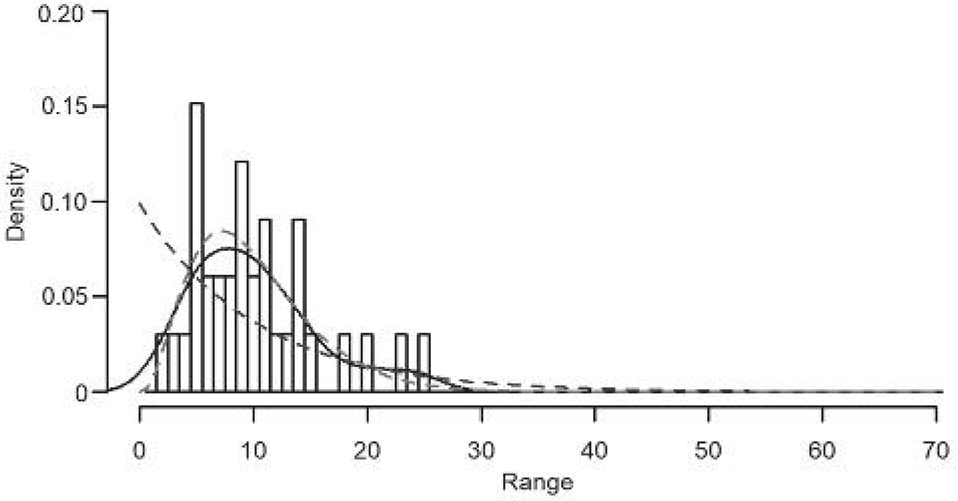

Appendix 1 Figure 1. Delay distribution from hospitalization to death, China, 2020. Gamma (dark, dashed), exponential distribution (light black, dashed) and kernel density distribution (black, full) were fitted to the available observed data (sample size 33 patients).

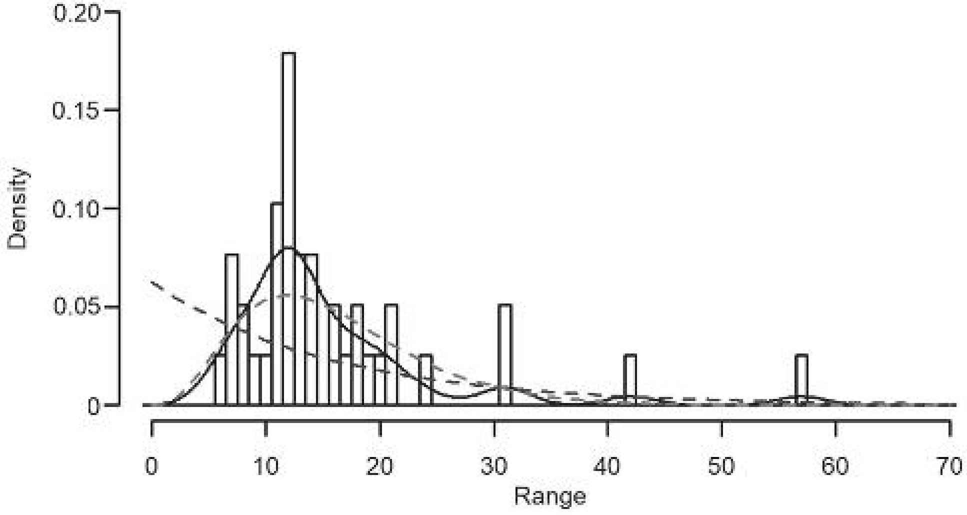

Appendix 1 Figure 2. Delay distribution from illness onset to death, China, 2020. Gamma (dark, dashed), exponential distribution (light black, dashed) and kernel density distribution (black, full) were fitted to the available observed data (sample size 39 patients).
